# Initial Physiological and Molecular Adjustments Underpin Salinity Tolerance During Wheat Germination and Early Seedling Development

**DOI:** 10.3390/plants15111593

**Published:** 2026-05-22

**Authors:** Murat Aycan

**Affiliations:** 1 Laboratory of Biochemistry, Institute for Social Innovation and Cooperation, Niigata University, Niigata 950-2181, Japan; murataycan@agr.niigata-u.ac.jp; 2 Department of Field Crops, Faculty of Agriculture, Ankara University, 06110 Ankara, Türkiye

**Keywords:** oxidative stress, proline metabolism, antioxidant enzymes, transcriptional regulation, abiotic stress markers

## Abstract

Global warming and associated environmental changes are reducing arable land and intensifying salinization risks, posing growing threats to food security. Soil salinity is an increasing threat to agricultural productivity worldwide, particularly in arid and semi-arid areas. Wheat (*Triticum aestivum* L.) is one of the most important and widely cultivated cereal crops for human consumption and livestock feed. However, with increasing water scarcity and the incidence of salt-affected lands, wheat productivity is increasingly affected by salinity. Previous studies have investigated salinity tolerance mechanisms mainly at the seedling and reproductive stages of wheat; however, comparatively fewer studies integrate rapid biochemical and physiological responses during the first hours of germination stress exposure together with transcriptional analyses during early seedling establishment, even though this stage is critical for stand establishment. Here, we evaluated early physiological and transcriptional responses of salt-tolerant, moderate, and sensitive wheat cultivars exposed to 0 or 150 mM NaCl during germination and the early seedling stage. Tolerant and sensitive cultivars showed contrasting germination performance under salinity. Physiological analysis showed that salt-tolerant cultivars exhibited higher proline accumulation and higher antioxidant enzyme activities (CAT, SOD, and GR), while maintaining lower MDA levels under salinity compared with sensitive cultivars. Notably, tolerant cultivars showed marked upregulation of *TaHKT1;4*, *TaP5CS*, *TaMYB*, and *TaDHN* genes associated with ion homeostasis, osmoprotectant metabolism, and stress-responsive regulation. These responses represent integrated early-stage biochemical, physiological, and transcriptional indicators of salinity responsiveness rather than direct predictors of final yield performance.

## 1. Introduction

Soil salinity is one of the most destructive abiotic stress factors that limits agricultural productivity worldwide. More than 20% of irrigated lands are currently salt-affected, and this area is projected to expand further under global climate change scenarios and human activities. High soil salinity significantly affects crop yield losses, including corn, cotton, and wheat, costing approximately US$73.3 billion yearly [1]. It is projected that high salinity will affect nearly 50% of the total arable agricultural land by 2050 [2]. Wheat (*Triticum aestivum* L.), the staple food for more than 2.5 billion people, is particularly vulnerable because it is frequently cultivated in arid and semi-arid regions where salinity is prevalent [3]. Developing salt-tolerant wheat cultivars is therefore a strategic priority for global food security. Although genetic variability for salt tolerance exists within bread wheat and its relatives, the underlying physiological and molecular mechanisms remain complex. They are strongly influenced by the developmental stage at which stress occurs [4].

Salinity tolerance is a complex trait involving numerous small molecular pathways and regulatory components [5]. The early stages of salt stress cause an osmotic imbalance, which is more pronounced than ion toxicity, leading to decreased water uptake and altered metabolic processes, as well as reduced chlorophyll production [6,7]. Plasma membrane proteins and genes, such as water channels and ion transporters, modulate Na^+^ and K^+^ balance during salt stress [8,9]. In wheat, HKT transporters such as *TaHKT1;4* contribute to Na^+^ transport and retrieval from the xylem, thereby contributing to processes associated Na^+^ transport and ion homeostasis [10,11]. Additionally, *TaDHN* plays a role in osmotic balance and antioxidant defense [12]. Additional genes associated with stress, such as *TaPTF1*, *TaSRG*, *TaSC*, *TaPIMP1*, *TaMIP*, *TaMYB*, and *TaGSK1*, are implicated in transcriptional regulation, ABA signaling, and stress-induced signaling pathways [12]. In contrast, *TaP5CS* plays a critical role in proline biosynthesis and osmotic adjustment [12,13]. Under saline conditions, Na^+^-induced membrane depolarization promotes K^+^ efflux through outward-rectifying channels, leading to reduced cellular K^+^ levels and subsequent oxidative stress, lipid peroxidation, and membrane damage [14]. Tolerant cultivars mitigate these effects via higher osmoregulation, reactive oxygen species detoxification, and ion exclusion [15]. Lower lipid peroxidation, such as malondialdehyde (MDA) content; higher proline accumulation [16,17], and increased activities of ROS-scavenging enzymes, such as catalase (CAT), superoxide dismutase (SOD), and glutathione reductase (GR), are commonly recognized indicators of salinity tolerance in plants [18,19,20]. Understanding the molecular mechanisms of salinity tolerance is crucial for identifying limitations in sensitive cultivars and maximizing benefits in tolerant cultivars.

Recent research on wheat salinity tolerance has focused on the reproductive stages, where traits such as ion concentration, osmotic balance, and agronomic and yield-related characteristics have been investigated [21,22]. However, salinity stress strongly limits germination and seedling establishment, which are among the most sensitive stages of development [23]. Despite the importance of germination and the seedling stage, these early developmental phases are less frequently integrated into comprehensive physiological and molecular analyses compared with agronomic traits. Understanding of the early responses to salinity stress during germination may provide a cost- and time-effective approach for identifying salinity-tolerant cultivars before long-term growth experiments. By focusing on the germination stage, which is an essential stage for salinity tolerance, we may gain insight into the development of salinity tolerance from the beginning and its potential application in breeding strategies [24]. Although salinity effects during wheat germination have been widely examined, most studies focus on single traits or later developmental stages, whereas integrated analyses capturing the immediate biochemical responses during early germination together with transcriptional responses during early seedling establishment remain limited. The focus of such early-stage analyses is not to predict final grain yield but to identify early physiological and molecular responses that distinguish stress-sensitive and stress-tolerant cultivars. In addition to conventional physiological screening approaches, recent genomic and modern biotechnological strategies, including transcriptomics, genome editing, precision breeding, and molecular-assisted selection, are increasingly being applied to improve salinity tolerance in crops by accelerating the identification of stress-responsive genes and regulatory pathways [25].

Unlike our previous studies, which primarily focused on seedling-stage salt exposure and physiological characterization of individual salinity-related pathways. This study integrates the rapid biochemical and physiological responses and early transcriptional profiling of wheat cultivars to salinity stress during germination and early seedling development. Six wheat cultivars previously classified as tolerant, moderate, or sensitive were exposed to control and salt treatments, and their responses were examined at both the immediate (1, 3, and 6 h) and early seedling (7 d) stages. To achieve this, we combined germination traits with biochemical markers of oxidative stress (MDA), osmotic adjustment (proline), and antioxidant defense (CAT, SOD, and GR), together with the expression of salinity-related genes (*TaPTF1*, *TaDHN*, *TaSRG*, *TaSC*, *TaPIMP1*, *TaMIP*, *TaHKT1;4*, *TaGSK1*, *TaP5CS*, and *TaMYB*). Multivariate, random forest, and machine learning approaches were utilized to integrate these datasets and identify the key traits associated with early salinity responsiveness in wheat cultivars. These findings provide integrated insight into the temporal coordination of physiological, biochemical, and transcriptional responses associated with early salinity responsiveness in wheat rather than identifying entirely new salinity-responsive pathways.

## 2. Results

### 2.1. Early Response of Wheat Cultivars to Salinity Stress

**Figure 1:**
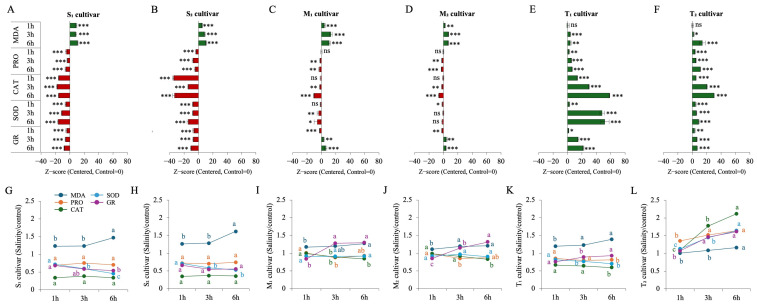
Early enzymatic antioxidant responses and their dynamics during seed germination under salt stress. Z-score standardized differences (stress vs. control) of malondialdehyde (MDA), proline (PRO), catalase (CAT), superoxide dismutase (SOD), and glutathione reductase (GR) activities at 1, 3, and 6 h after germination start in (**A**) sensitive-1 (S1), (**B**) sensitive-2 (S2), (**C**) moderate-1 (M1), (**D**) moderate-2 (M2), (**E**) tolerant-1 (T1), and (**F**) tolerant-2 (T2) cultivars. Relative temporal changes in enzymatic activities (CAT, SOD, GR) and stress markers (MDA, PRO) are expressed as fold change in salt over control at 1, 3, and 6 h after germination start in (**G**) sensitive-1 (S_1_), (**H**) sensitive-2 (S_2_), (**I**) moderate-1 (M_1_), (**J**) moderate-2 (M_2_), (**K**) tolerant-1 (T_1_), and (**L**) tolerant-2 (T_2_) cultivars. Positive values (green color) indicate higher accumulation/activity under stress compared with the corresponding control, while negative values (red color) indicate reductions in Z-score. Bars represent mean ± SE (*n* = 5). Different color letters and asterisks denote significance levels (ns = non-significant, * *p* < 0.05, ** *p* < 0.01, *** *p* < 0.001; two-way ANOVA followed by Tukey’s HSD test).

To evaluate the immediate effects of salinity, biochemical markers were analyzed at 1, 3, and 6 h following exposure to control (0 mM NaCl) and salinity (150 mM NaCl) stress after 3 days of germination in salt-sensitive cultivars Sonmez-01 (S_1_) and Esperia (S_2_), moderately tolerant cultivars Bezostoja (M_1_) and Gerek-79 (M_2_), and salt-tolerant cultivars Ikizce-96 (T_1_) and Demir-2000 (T_2_). Results showed that salinity activated a distinct cultivar-dependent salinity response in oxidative stress at the germination as immediate salinity response of salt stress. Malondialdehyde (MDA) content significantly increased from 1 h and remained elevated through 6 h in sensitive cultivars (S_1_, S_2_), indicating that rapid lipid peroxidation initiates under salinity stress (Figure 1A,B and Figures S1A, S2A and S3A). Moderate cultivars (M_1_, M_2_) showed a slight but significant increase in MDA content in the early hours (Figure 1C,D and Figures S1A, S2A and S3A). Although lipid peroxidation is highly prevalent, the level was lower than in sensitive cultivars, suggesting a partial protective capacity. The tolerant cultivars (T_1_, T_2_) exhibited the lowest MDA accumulation compared to sensitive and moderate cultivars under salt stress (Figure 1E,F and Figures S1A, S2A and S3A), although a gradual increase was observed over time, particularly at 3 h and 6 h, indicating improved but not complete protection of membrane stability under salinity conditions.

Proline (PRO) accumulation was observed to be lower in S_1_ and S_2_ cultivars compared to the moderate and tolerant cultivars in the first hours of salinity. Still, it was not significantly different at 3 and 6 h under control conditions. Salinity significantly reduced proline accumulation in sensitive cultivars (Figure 1A,B and Figures S1B, S2B and S3B). The PRO accumulation of moderate cultivars was not significantly altered 1 h after salinity stress; however, 3 h after salinity stress, PRO content was considerably reduced in the moderate cultivars. The PRO accumulation was decreased slightly in moderate cultivars after 6 h of salinity stress application (Figure 1C,D and Figures S1B, S2B and S3B). Notably, T_1_ and T_2_ cultivars showed a significant increase in PRO accumulation from 1 h to 6 h of salinity stress compared to the control condition (Figure 1E,F and Figures S1B, S2B and S3B). This rapid PRO accumulation reflects efficient osmoprotectant activity in tolerant cultivars.

Catalase (CAT) activity exhibited distinct cultivar-dependent variations in the initial hours of salinity stress. In S_1_ and S_2_ cultivars, CAT activity showed a significant reduction at 1 h, remaining significantly lower than that of moderate and tolerant cultivars at all time points (Figure 1A,B and Figures S1C, S2C and S3C). The CAT activity was recorded as almost the same in M_1_ and M_2_ cultivars at 1 h after salinity stress under both control and salinity stress conditions. After 3 h of salinity stress, the CAT activity of moderate cultivars began to show a significant reduction, and this reduction was observed 6 h after the onset of salinity stress in moderate cultivars (Figure 1C,D and Figures S1C, S2C and S3C). In contrast, T_1_ and T_2_ cultivars exhibited significant induction of CAT activity as early as 1 h, with significantly higher activity sustained through 3 h and 6 h of salinity stress conditions (Figure 1E,F and Figures S1C, S2C and S3C).

The superoxide dismutase (SOD) activity showed a significant reduction in sensitive cultivars under salinity stress for 1 h onward, and this reduction persisted through the 3 h and 6 h time points (Figure 1A,B and Figures S1D, S2D and S3D). In moderate cultivars, M_1_ did not exhibit a significant change in SOD activity at 1 h under salinity stress; however, SOD activity slightly decreased after 3 h and remained reduced at 6 h. In contrast, M_2_ exhibited an overall lower SOD response under salinity stress across all measured time points (Figure 1C,D and Figures S1D, S2D and S3D). Tolerant cultivars exhibited higher SOD activity under salinity stress compared with the control conditions from 1 h to 6 h of exposure. Additionally, the tolerant cultivars showed the highest SOD activity among all tested cultivars under salinity stress (Figure 1E,F and Figures S1D, S2D and S3D).

The glutathione reductase (GR) activity was negatively affected by salinity stress application in sensitive cultivars, and it was significantly reduced at 1, 3, and 6 h of stress exposure (Figure 1A,B and Figures S1E, S2E and S3E). A similar pattern was observed in the moderate cultivar at 1 h salinity stress application, where GR activity significantly reduced. Notably, after 3 h of salinity stress, GR activity increased slightly in moderate cultivars compared to control conditions, and GR was measured considerably higher in moderate cultivars under salinity stress for 6 h compared to control conditions (Figure 1C,D and Figures S1E, S2E and S3E). The tolerant cultivars showed significantly higher GR activity at all 1, 3, and 6 h salinity stress application periods compared to the control and other sensitive and moderate cultivars (Figure 1E,F and Figures S1E, S2E and S3E).

### 2.2. The Tolerant Cultivars Showed Higher Germination Success Under Stress Conditions

**Figure 2:**
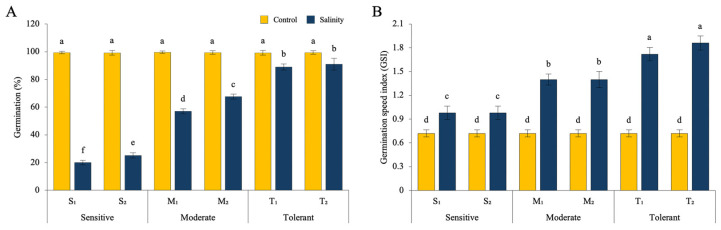
Effects of salt stress on germination traits in wheat genotypes. (**A**) Germination percentage (GP) of sensitive (S_1_, S_2_), moderate (M_1_, M_2_), and tolerant (T_1_, T_2_) cultivars under control (yellow, 0 mM NaCl) and salt stress (blue, 150 mM NaCl) conditions. Tolerant cultivars maintained significantly higher germination compared with sensitive cultivars. (**B**) Germination speed index (GSI) under control and salt stress. Different letters above bars indicate significant differences among cultivar × treatment combinations (two-way ANOVA followed by Tukey’s HSD, *p* < 0.05). Data are mean ± SE (n = 5).

Salinity stress significantly reduced the germination percentage (GP) of all wheat cultivars, revealing apparent differences among the tolerant, moderate, and sensitive groups (Figure 2). All cultivars exhibited high GP, ranging from 95% to 100%, with no significant differences observed among the groups in control conditions. When sensitive cultivars (S_1_ and S_2_) were exposed to 150 mM NaCl, their growth performance dropped significantly, dropping below 40% after 3 days (Figure 2A). Moderate cultivars (M_1_ and M_2_) showed lower germination rates when exposed to salt stress. However, their germination rates were still much higher than those of sensitive lines. The tolerant cultivars (T_1_ and T_2_) had the highest germination rate under salinity, over 80%, and there was no significant difference between them and their control values.

The germination speed index (GSI) showed a clear response to salinity stress conditions. Sensitive cultivars exhibited significantly higher GSI values under salinity compared to the control, indicating delayed germination and less efficient germination. Moderate and tolerant cultivars also showed increased GSI values under salinity stress, although the magnitude of the response varied among cultivars (Figure 2B). In the present study, higher GSI values reflect delayed germination timing because the index was calculated using a weighted germination timing formula. Therefore, these findings indicate that salinity markedly delayed germination in wheat, while tolerant cultivars maintained relatively faster and more stable germination compared with sensitive and moderate cultivars.

### 2.3. Germination Performance Affects the Seedling Physiology, Photosynthetic Pigment Accumulation and Biochemical Traits

**Figure 3:**
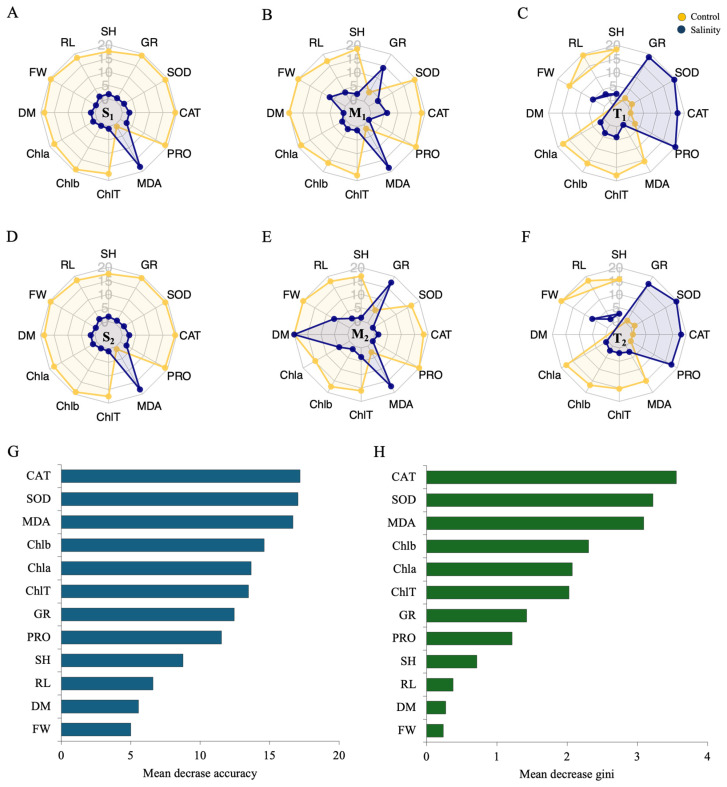
Multivariate profiling of physiological and biochemical responses in wheat genotypes under salt stress. Radar plots showing the relative performance of (**A**) sensitive-1 (S_1_), (**B**) moderate-1 (M_1_), (**C**) tolerant-1 (T_1_), (**D**) sensitive-2 (S_2_), (**E**) moderate-2 (M_2_), (**F**) tolerant-2 (T_2_) cultivars across multiple traits. Values are expressed as standardized scores (Z-scores) relative to control, with yellow areas indicating control (0 mM NaCl) and the blue regions indicating salt stress (150 mM). Random forest analysis ranking trait importance for discriminating cultivars under control and salt stress. Importance was assessed using (**G**) mean decrease accuracy and (**H**) mean decrease Gini index. Malondialdehyde (MDA), proline (PRO), catalase (CAT), superoxide dismutase (SOD), glutathione reductase (GR), chlorophyll a (Chla), chlorophyll b (Chlb), total chlorophyll (ChlT) pigments, shoot height (SH), root length (RL), fresh weight (FW), dry matter (DM).

To integrate the multidimensional responses, radar plots were generated for each cultivar (Figure 3A–F). The seedlings exposed to salinity stress after germination showed different salinity tolerance responses among the tested cultivars. Salinity stress significantly reduced shoot height (SH) in all cultivars. Sensitive cultivars were decreased by around 50% of SH. Moderate cultivars lose SH by around 20%, and tolerant cultivars reduce SH by just 10%. Under salinity stress, the tolerant cultivars showed higher SH than the other tested sensitive and moderate cultivars (Figure 3A–F and Figure S4A upper panel). A similar pattern was observed in root length (RL). Salinity stress reduced RL by approximately 50%, 25%, and 14% in sensitive, moderate, and tolerant cultivars, respectively. The tolerant cultivars have lower RL reduction and higher RL under both control and salinity stress conditions compared to the sensitive and moderate cultivars (Figure 3A–F and Figure S4A, lower panel).

The salinity stress effects were reflected in plant fresh weight (FW) and dry matter (DM) traits. The FW was significantly negatively affected by salinity stress. The FW was reduced by 18% to 64% among cultivars under salt stress compared to control conditions. The tolerant cultivars showed higher FW under control and salinity stress conditions compared to sensitive and moderate cultivars (Figure 3A–F and Figure S4B). These results were reflected in DM values in all tested cultivars. The DM was significantly reduced by 15% to 71% among tested cultivars under salinity stress compared to control conditions. Still, the highest DM was recorded in tolerant cultivars under both control and salinity stress conditions (Figure 3A–F and Figure S4C). Notably, several physiological and biochemical traits also showed significant baseline differences among sensitive, moderate, and tolerant cultivars under control conditions, indicating inherent cultivar-dependent variation independent of salinity stress treatment.

The photosynthetic pigments (Chla, Chlb, and ChlT) were highly affected by salinity stress in all cultivars. The Chla was significantly reduced by 10% to 69% in the tested wheat seedlings. Under control conditions, the tolerant cultivars showed higher Chla content compared to the sensitive and moderate cultivars, but the sensitive and moderate cultivars exhibited almost similar Chla content. Under salt stress conditions, the tolerant cultivars showed lower Chla reduction and higher Chla content among all cultivars (Figure 3A–F and Figure S4D). The Chlb content showed a similar pattern to the Chla content. Chlb content was reduced by 15% to 70% in all wheat seedlings under salinity stress compared to control conditions. The higher reduction and lower Chlb content were observed in the sensitive cultivars, and the lowest reduction and highest Chlb content were recorded in the tolerant cultivars (Figure 3A–F and Figure S4E). The ChlT was also reduced by 14% to 61% under salinity stress among the tested cultivars. The lower reduction and higher ChlT content were observed in the tolerant cultivars, as were the same Chla and Chlb content (Figure 3A–F and Figure S4F).

The biochemical analysis at the seedling stage revealed that sensitive wheat cultivars showed higher MDA content, and salinity stress increased MDA accumulation by 30% to 90% in the sensitive and moderate cultivars. In contrast, the MDA content of tolerant cultivars was significantly reduced by around 30% under salinity stress compared to control conditions and the other tested sensitive and moderate cultivars (Figure 3A–F and Figure S5A). Conversely, osmoprotectant PRO accumulation was significantly reduced in sensitive and moderate cultivars by 18% to 65%, and tolerant cultivars increased dramatically by 91% and 125% under salinity stress compared to control conditions (Figure 3A–F and Figure S5B). The CAT activity at the seedling stage was significantly reduced by 14% to 74% in moderate and sensitive cultivars, but it was significantly increased by 53% and 90% in the tolerant cultivars (Figure 3A–F and Figure S5C). The SOD activity of moderate and sensitive cultivars was significantly decreased by 25% to 71% under salinity stress, whereas tolerant cultivars increased SOD activity by 46% and 52% under the same conditions (Figure 3A–F and Figure S5D). Additionally, GR activity also showed a different pattern with CAT and SOD activities, which showed that sensitive cultivars reduced GR activity by around 60% under salinity conditions; however, moderate and tolerant cultivars significantly increased GR activity by 13% to 50% under salinity stress (Figure 3A–F and Figure S5E). Analysis of variance (ANOVA) confirmed highly significant effects of genotype (Cv), environment (E), and their interaction (Cv × E) for almost all traits studied (Table 1). Early biochemical markers such as MDA, proline, CAT, SOD, and GR showed strong genotype- and treatment-specific responses, with significant Cv × E interactions at all measured time points (Table S2).

### 2.4. Trait Importance Ranking by Random Forest Analysis

The random forest analysis was performed to determine the most informative traits for salinity stress tolerance. A random forest model was used to rank traits by their relative contribution to cultivar separation under salinity stress. The antioxidant traits (CAT and SOD) and MDA consistently ranked as the most informative traits in this exploratory analysis. The morphological (SH, RL, FW, and DM) traits contributed a lower level to cultivar classifications, indicating that morphological characteristics are not sufficient for early screening for salinity tolerance (Figure 3G,H). These findings suggest that the balance between protective and damage-related markers may be associated with salinity tolerance responses among cultivars.

### 2.5. Trait Separation by Principal Component and Hierarchical Clustering Analyses

**Figure 4:**
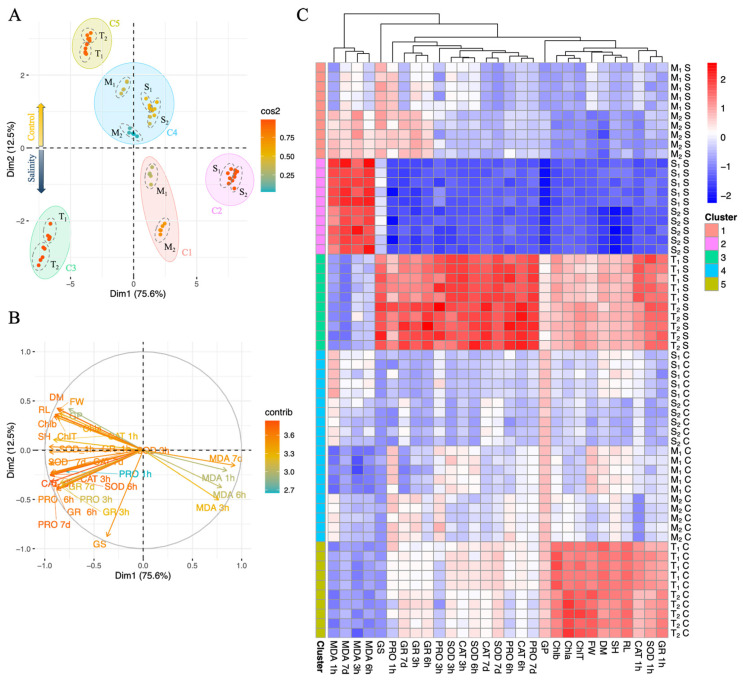
Multivariate clustering and correlation patterns of wheat seedlings under salt stress. (**A**) Principal component analysis (PCA) score plot showing clear separation among sensitive (S_1_, S_2_), moderate (M_1_, M_2_), and tolerant (T_1_, T_2_) cultivars under control (0 mM NaCl) and salt stress (150 mM NaCl) conditions. Ellipses indicate 95% confidence intervals. (**B**) PCA biplot displaying the contribution of physiological and biochemical traits to cultivar separation. Antioxidant enzymes (CAT, SOD, GR) and oxidative stress markers (MDA, PRO) strongly influenced the clustering of tolerant versus sensitive lines. (**C**) Hierarchical clustering heatmap of Z-score standardized traits across all cultivars and treatments. Red and blue represent high and low relative values, respectively. Clustering highlights distinct stress-response profiles, with tolerant cultivars forming a separate cluster driven by enhanced antioxidant activity and pigment stability, while sensitive cultivars clustered with higher oxidative damage (MDA) and reduced growth parameters.

Principal component analysis (PCA) was conducted to illustrate the overall trait variation among wheat cultivars under both control conditions and salinity stress conditions (Figure 4A,B). The initial two dimensions (Dim1 and Dim2) accounted for 75.6% and 12.5% of the total variance, respectively, resulting in an overall explanation of 88.1%. The PCA score plot (Figure 4A) demonstrated distinct separation among tolerant (T_1_, T_2_), moderate (M_1_, M_2_), and sensitive (S_1_, S_2_) cultivars in response to control and salinity stress. Tolerant cultivars under control were clustered in C3 and, under salinity stress, were clustered in C5. Moderate and sensitive cultivars were grouped in the same cluster (C4) under control conditions; however, under salinity stress, sensitive and tolerant cultivars were clustered in separate clusters as C2 and C1, respectively. The PCA biplot (Figure 4B) indicated that antioxidant enzymes (CAT, SOD, and GR), proline, and chlorophyll pigments (Chla, Chlb, and ChlT) had a positive contribution, significantly affecting the clustering of tolerant cultivars. The MDA demonstrated a significant negative loading, correlating closely with sensitive cultivars, and the antioxidant enzymes and biochemical traits showed a significant positive loading with tolerant cultivars. The morphological traits (SH, RL, FW, and DM) contributed minimally to the variance along either axis, indicating their limited role in differentiating tolerance at this stage.

Hierarchical clustering analysis (HCA) also confirmed the cultivar-specific PCA model (Figure 4C). Employing Pearson correlation as the similarity metric and Euclidean distance for dissimilarity, the heatmap clearly clustered cultivars (tolerant, moderate, and sensitive) and conditions (control and salinity). Moderate cultivars were clustered in C1, sensitive cultivars were grouped in C2, and tolerant cultivars were clustered in C3 under salinity stress. The C4 represents sensitive and moderate cultivars, and the C5 includes tolerant cultivars under control conditions. Additionally, heatmap clustering separated tolerant cultivars (T_1_ and T_2_) as a distinct tolerant cluster, characterized by higher antioxidant activity, proline accumulation, and pigment stability. Sensitive cultivars (S_1_ and S_2_) were grouped due to elevated MDA levels and diminished antioxidant responses, whereas moderate cultivars (M_1_ and M_2_) occupied an intermediate position between the tolerant and sensitive categories.

The results from PCA and HCA indicate that biochemical traits, particularly antioxidant enzyme activities, proline content, and chlorophyll pigments, are the main factors influencing cultivar separation under salinity, while growth parameters contribute minimally. The analyses present compelling evidence that tolerant wheat lines exhibit coordinated antioxidant and osmoprotectant responses, differentiating them from sensitive cultivars under early salinity stress.

### 2.6. Expression Patterns of Salinity-Responsive Genes in the Tested Cultivars

To further explore the molecular basis of salinity tolerance, the expression of ten stress-related genes (*TaPTF1*, *TaDHN*, *TaSRG*, *TaSC*, *TaPIMP1*, *TaMIP*, *TaHKT1;4*, *TaGSK1*, *TaP5CS*, and *TaMYB*) was analyzed in tolerant, moderate, and sensitive wheat cultivars under control and salt stress conditions. Sensitive cultivars (S_1_ and S_2_) showed a gene-dependent response to salinity. In sensitive cultivars, *TaHKT1;4* expression level decreased by approximately 22–35% under salinity stress compared to the control, whereas *TaP5CS* expression was reduced by nearly 18–40%. In contrast, *TaMYB* and *TaPTF1* showed limited induction (approximately 5–10%) or remained unchanged under salinity stress (Figure S6).

Moderate cultivars (M_1_ and M_2_) exhibited an intermediate expression response. *TaHKT1;4* and *TaP5CS* were induced by 15–35%, and stress-related transcription factors like *TaSRG*, *TaSC*, and *TaMYB* were increased by 20–45% under salinity stress. The level of induction was still much lower than what was seen in tolerant cultivars, even though it was stronger than in sensitive lines (Figure S6).

The tolerant cultivars, on the other hand (T_1_ and T_2_), mounted a robust transcriptional response across nearly all genes. For example, when compared to the control conditions, the expression of *TaHKT1;4* increased by 110% and 135%, while the expression of *TaP5CS* showed a remarkable induction of 145% and 172%. Similar to *TaMYB*, *TaDHN* was elevated by 95–130%, which is indicative of a significant activation of regulatory mechanisms. In tolerant lines, the expression of other stress-associated genes, such as *TaPTF1*, *TaSRG*, *TaSC*, *TaPIMP1*, and *TaGSK1*, was consistently increased by 60–100%. In contrast, the expression of *TaMIP* increased by approximately 40–65% under salinity stress (Figure S6).

**Figure 5:**
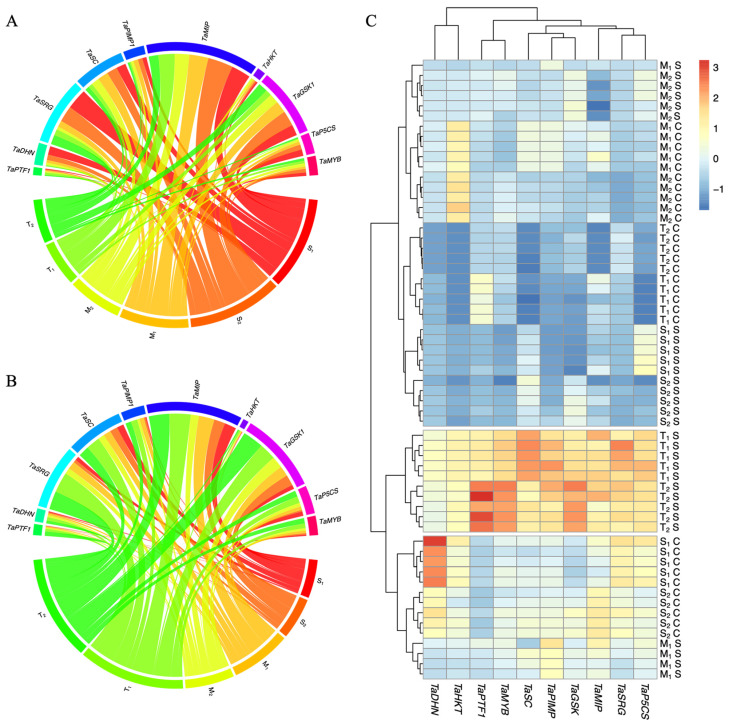
Expression patterns of stress-responsive genes in wheat cultivars under control and salinity conditions. Circular diagrams illustrating the relative associations between wheat cultivars and salinity-responsive genes (*TaPTF1*, *TaDHN*, *TaSRG*, *TaSC*, *TaPIMP1*, *TaMIP*, *TaHKT1;4*, *TaGSK1*, *TaP5CS*, and *TaMYB*) expression patterns under (**A**) control and (**B**) salinity stress conditions. The ribbons represent relative associations between cultivars and gene expression patterns, with thicker connections indicating stronger relative associations. (**C**) Heatmap of relative expression levels of the ten genes in tolerant (T_1_, T_2_), moderate (M_1_, M_2_), and sensitive (S_1_, S_2_) wheat cultivars under control (C) and salinity (S) stress conditions. Red indicates high expression and blue indicates low expression patterns.

The chord diagram visualizations (Figure 5A,B) and expression heatmap (Figure 5C) further illustrated the distinct expression–pattern relationship among cultivars and salinity-responsive genes. Interactions were infrequent under controlled conditions, indicating basal activity. Under salinity stress, though, tolerant cultivars showed a lot of co-expression between *TaHKT1;4*, *TaP5CS*, *TaMYB*, and *TaDHN*. This shows how important these genes are for coordinating stress defense. Sensitive lines exhibited an absence of such connectivity, whereas moderate lines displayed partial yet diminished associations.

These results demonstrate that tolerant cultivars exhibit a coordinated transcriptional response, characterized by substantial increases (≥100%) in key functional and regulatory genes. In contrast, sensitive cultivars do not activate these pathways as well. The robust induction of ion transport (*TaHKT1;4*), osmoprotectant-related (*TaP5CS*), and regulatory genes (*TaMYB* and *TaDHN*) was associated with enhanced physiological performance in tolerant lines in saline conditions.

## 3. Discussion

This study provides integrated insights into the physiological and molecular response of wheat cultivars to salinity stress during the transition from germination to early seedling establishment. The early salinity stress exposure results revealed the primary difference in oxidative stress and lipid peroxidation between tolerant and sensitive cultivars during germination, which may contribute to improved seedling establishment under salinity. Sensitive cultivars exhibited rapid and significant elevations in MDA within one hour of exposure, with this increase sustained throughout the initial time course. MDA is a lipid peroxidation and ROS-induced membrane damage indicator in plants, and higher MDA levels have been linked to salt stress sensitivity [14,19,26]. The observations showed that tolerant cultivars accumulated comparatively lower MDA levels under saline conditions despite gradual increases over time, indicating enhanced protection of their cellular membranes against oxidative damage. Furthermore, the intensity of early oxidative stress may serve as an indicator of cultivar-level salinity responsiveness. Similar results have been observed in rice [27] and barley [28], indicating that tolerant cultivars accumulated markedly lower levels of MDA compared to sensitive lines under saline conditions.

These biochemical responses were associated with reduced oxidative damage under salinity stress. In the first few hours of salinity stress, tolerant lines exhibited rapid increases in CAT, SOD, and GR activities. Moderate lines showed intermediate responses, and sensitive lines showed lower antioxidant defenses. The early activation of these enzymes likely contributes to limiting oxidative damage during initial stress exposure, although such responses may reflect stress adjustment rather than long-term tolerance outcomes. Numerous studies have identified antioxidant enzyme activity as a significant factor influencing salt tolerance in wheat, barley, and rice [28,29,30,31]. In our analysis, antioxidant enzyme activities reflected cultivar-specific oxidative responses and were among the most informative variables distinguishing stress-sensitive and stress-tolerant responses at early developmental stages.

Proline accumulation differentiated cultivar responses during early salinity exposure, reflecting short-term osmotic and redox adjustment rather than direct predictors of yield-level tolerance. In our experiment, proline accumulation significantly increased in tolerant cultivars from the early hours of salinity stress. However, proline accumulation was reduced or increased only slightly in sensitive and moderate cultivars, respectively. Previous research also indicated that proline is one of the essential indicators for salinity stress tolerance because of its protective role against osmotic stress, stabilizing proteins and membranes, and removing ROS in salt stress-exposed plants [12,17,32]. Our findings suggest that proline biosynthesis, facilitated by the *TaP5CS* gene, is elevated within hours of stress in tolerant lines, providing an early defense against osmotic and oxidative stress. Additionally, the strong correlation between the biochemical results and gene expression patterns confirms the essential role of proline metabolism in early salinity tolerance. Similar links between P5CS induction and proline accumulation have been seen in rice [32], soybean [33], and Arabidopsis [34].

We examined the effects of increased enzymatic and osmoprotectant activity during the early response to salinity stress on the transcriptional changes during the early seedling establishment period. We observed that tolerant cultivars exhibited differential activation of several stress-responsive genes, which involved ion transport, osmoprotection, and regulatory function. The higher and stronger response was recorded in the *TaHKT1;4, TaP5CS*, *TaMYB*, and *TaDHN* genes. The *TaHKT1;4* gene is associated with sodium transport and processes related to ion homeostasis under salinity stress, primarily through Na^+^ retrieval and transport regulation in vascular tissues [35]. *TaP5CS* encodes a key enzyme involved in proline biosynthesis, contributing to osmotic adjustment and cellular redox balance during stress conditions [17]. *TaMYB* transcription factors function as regulatory components involved in ABA-related and salt stress-responsive signalling pathways that coordinate downstream defense responses [36]. In contrast, *TaDHN* proteins are mainly localized in cellular membranes and the cytoplasm, where they contribute to membrane stabilization, protein protection, and osmotic stress tolerance under saline conditions [37,38]. Other tested genes, *TaPTF1*, *TaSRG*, *TaSC*, *TaPIMP1*, and *TaGSK1*, are involved in signaling and regulation and were also activated in tolerant cultivars under salt stress conditions [12,37,39,40,41]. However, sensitive cultivars exhibited limited or reduced expression of several stress-responsive genes under salinity stress, indicating weaker transcriptional responsiveness compared with tolerant cultivars. Moderate cultivars showed intermediate expression responses, with slight induction of several stress-responsive genes under salinity stress. These results indicate limited activation of regulatory genes and a restricted activity of protective effectors against salinity stress. Previous studies have shown that HKT transporters [42], dehydrins [43], and stress-induced MYB transcription factors [44] are important regulators of salinity tolerance in wheat and other cereals. However, previous transcriptomic and physiological studies have also shown that salinity tolerance responses can vary substantially depending on genotype, developmental stage, stress duration, and experimental conditions, indicating that salinity adaptation involves highly dynamic and multifactorial regulatory mechanisms [21,22,45].

The chord diagram visualization revealed distinct relationships between wheat cultivars and stress-responsive gene expression patterns under control and salinity conditions. Under control conditions, relative associations between cultivars and stress-responsive genes were comparatively limited. Under salinity stress, however, tolerant cultivars showed stronger relative associations with several genes, particularly *TaHKT1;4*, *TaP5CS*, *TaMYB*, and *TaDHN*. In contrast, sensitive cultivars displayed comparatively weaker association patterns with stress-responsive genes. These observations suggest that tolerant cultivars exhibit stronger transcriptional responsiveness under salinity stress conditions. Similar stress-responsive transcriptional patterns under salinity stress have also been reported in previous transcriptomic studies in wheat [46] and rice [45].

The machine learning tools, such as multivariate analysis and random forests, are powerful tools for classifying and understanding multidimensional biological data [47,48]. PCA clearly separated tolerant, moderate, and sensitive cultivars according to the integration of physiological and biochemical traits. PCA showed that antioxidant enzymes, proline, and pigments contributed to the clustering of tolerant cultivars, while MDA exhibited a strong correlation with sensitive cultivars. Hierarchical clustering analysis identified specific clusters, validating the accuracy of the trait profiles. Although both M_1_ and M_2_ were classified as moderately tolerant cultivars, some physiological traits such as SOD activity and dry matter accumulation showed cultivar-specific variation between the two lines. This partial separation was also reflected in the PCA, suggesting that moderate salinity tolerance may involve heterogeneous physiological response patterns among cultivars. Random forest analysis provided an exploratory trait prioritization pattern associated with salinity responsiveness among cultivars. This indicated that antioxidant enzymes, proline, pigments, and MDA were the best traits for distinguishing between tolerant and sensitive cultivars. Recent applications in plants demonstrate the ability of machine learning to understand trait relations under abiotic stresses such as drought and salinity [49,50]. Because of the limited number of cultivars and the absence of cross-validation, the machine learning analyses in this study should be interpreted as exploratory rather than predictive. Although many of the physiological markers and stress-responsive genes evaluated in this study have previously been associated with salinity tolerance during vegetative and reproductive stages in wheat and other cereals, the present study specifically focuses on their rapid responsiveness during the transition from germination to early seedling establishment. Therefore, the novelty of this work lies not in the identification of new salinity-associated markers but in the integrated evaluation of early biochemical, physiological, transcriptional, and machine learning-based response patterns during the initial stages of salinity exposure.

Previous studies have shown that several of the physiological and molecular responses examined in this study, including antioxidant regulation, proline accumulation, ion transport, and stress-responsive transcription factors, also contribute to salinity tolerance during vegetative and reproductive stages in wheat and other cereals [12,17,30,42,43,44]. Therefore, the early responses observed here likely reflect components of broader salinity-response mechanisms operating throughout plant development rather than completely stage-specific processes. However, the rapid activation patterns detected during germination and early seedling establishment may provide useful early-stage signatures associated with salinity responsiveness before long-term developmental and yield-related effects become visible. Although these early markers cannot fully predict mature-plant performance under field conditions, they may support preliminary screening approaches for eliminating highly sensitive genotypes prior to extensive field evaluation.

These findings are especially significant from a breeding standpoint. Recent advances in genomics, precision breeding, transcriptomics, and genome-editing technologies such as CRISPR/Cas systems are increasingly contributing to the development of salt-tolerant crops by accelerating the identification and manipulation of stress-responsive genes and regulatory pathways [25]. In the past, choosing plants that can handle salty soil has often meant looking at how well they grow in the field, which is expensive, takes a lot of time, and is significantly affected by changes in the environment. Our research indicates that tolerance can be preliminarily inferred from traits assessed during the initial hours of germination. Assays for MDA, proline, antioxidant enzyme activity, and key stress-responsive genes such as *TaHKT1;4* and *TaP5CS* are cost-effective and suitable for repeated screening. This makes them perfect for early-stage screening traits. Integrating these markers into breeding programs could accelerate the identification of salt-tolerant cultivars, thereby reducing the need for extensive field experiments. These approaches align with the recent trend of using physiological markers and omics technologies in the cereal breeding process [51,52].

Importantly, the physiological and molecular responses characterized in this study should be interpreted as early-stage indicators of salinity responsiveness rather than direct determinants of final grain yield. Germination and early seedling stages represent a critical developmental window where stress perception, signaling, and protective mechanisms are rapidly established. While these early responses do not fully capture long-term field performance, they provide a robust and cost-effective framework for eliminating highly sensitive genotypes prior to resource-intensive field evaluations. A limitation of the present study is that ionic parameters such as Na^+^ and K^+^ accumulation were not directly quantified. Therefore, interpretations are restricted to early osmotic and oxidative responses rather than comprehensive ion homeostasis mechanisms. Future studies integrating ionic profiling with early biochemical and molecular markers will be essential to link early stress responses with long-term salinity tolerance.

## 4. Materials and Methods

### 4.1. Plant Material, Germination Conditions and Salinity Treatment

Sonmez-01 (S_1_) and Esperia (S_2_) were used as salt-sensitive cultivars, Bezostoja (M_1_) and Gerek-79 (M_2_) as moderately salt-tolerant cultivars, and Ikizce-96 (T_1_) and Demir-2000 (T_2_) as salt-tolerant cultivars (Table S3), based on our previous study [53]. Seeds were surface sterilized using 5% sodium hypochlorite for 10 min with continuous stirring, followed by three rinses with sterile distilled water [54]. Sterilized seeds were germinated on moist blotting paper in plastic Petri dishes. After 3 days of incubation in dark conditions at 23 °C, germination percentages (mean ± SD) were calculated when the emerging radicle had elongated to 3 mm. To determine early-stage salinity responses, we applied 150 mM NaCl, a concentration previously reported to cause approximately 50% yield reduction at later developmental stages [55,56]. For germination percentage and germination speed index traits, seeds were exposed to salinity stress from day 0. For early-stage response, germinated seeds were transferred to pots and irrigated with either distilled water (control, 0 mM NaCl) or a saline solution (150 mM NaCl). The salinity solution was applied daily to maintain a consistent salt concentration in the growth medium throughout the 7-day treatment period. Early physiological responses to salinity were measured at 1, 3, and 6 h after the initiation of salt treatment. These early sampling time points were selected to capture rapid oxidative and osmotic stress responses during the initial phase of salinity exposure, consistent with previous early-stage salinity studies in wheat and other cereals [45,57]. For early germination-stage biochemical analyses (1, 3, and 6 h), whole germinated seeds were collected as experimental samples. For 7-day seedling-stage physiological, biochemical, and gene expression analyses, shoot tissues were collected. For seedling establishment analyses, salinity treatment was continued for 7 days under controlled growth chamber conditions. After 3 days of germination, germinated seeds were transferred to 0.5 L pots filled with peat-based growing media (propagation substrate SF1, SuliFlor, Radviliškis, Lithuania) with the following characteristics: pH: 6, electrical conductivity (EC): 0.65, organic matter: 80%, and N–P_2_O_5_–K_2_O 14:16:18. Plants were grown in a controlled growth chamber under a 16 h light / 8 h dark photoperiod at 23 ± 1 °C, with a light intensity of approximately 200 µmol m^−2^ s^−1^ and relative humidity of 60–70%. Plants were exposed to salinity for 7 days. Seedling traits were measured at 7-day-old seedlings. Each biological replicate consisted of 20 germinated seeds for germination-stage analyses and 5 plants for seedling-stage physiological, biochemical, and gene-expression analyses. Biochemical analyses during the germination stage were performed using whole germinated seedlings collected at 1, 3, and 6 h after salinity exposure. In contrast, physiological, biochemical, and gene-expression analyses at the seedling stage were conducted using shoot tissues collected from 7-day-old seedlings continuously exposed to salinity stress. A pestle and mortar were used to turn snap-frozen shoots into a fine powder in liquid nitrogen. The samples were kept at −80 °C until they were used.

### 4.2. Physiological Analysis

The germination percentage (GP) was recorded on the third day. The germination speed index (GSI) was calculated as GSI = [(Number of seeds germinated on the day of counting) × (The day of the census)]/(Total number of germinated seeds) according to Ellis and Roberts [58]. Chlorophyll a (Chla), b (Chlb), and total chlorophyll (ChlT) content were recorded according to Curtis and Shetty [59]. Fresh leaves (50 mg) were mixed with 1.5 mL of ethanol and left to sit at 23 °C in the dark for two hours. A UVmini-1240 spectrophotometer (Shimadzu, Kyoto, Japan) was used to measure the absorbance at 650 and 665 nm.

Shoot height (SH) and root length (RL) were measured 7 days after salinity stress was applied. The fresh weight (FW) of whole seedlings (shoot + root) was recorded using a sensitive balance, and samples were dried in an oven set at 85 °C until they reached a steady weight. Dry matter (DM) was then recorded using a sensitive balance.

### 4.3. Biochemical Assays

The free proline (PRO) content was measured using the Bates et al. [60] method with minor modifications. Fresh leaf samples were homogenized in 10 mL of 3% sulfosalicylic acid and incubated at 4 °C for 24 h. After centrifuging the homogenate at 10,000× *g* at 25 °C for 5 min, the supernatant (1 mL) was reacted with 1 mL of ninhydrin reagent and 1 mL of glacial acetic acid in a test tube at 100 °C for 1 h. The reaction was stopped by immersing the tubes in an ice bath for 20 min. Proline was extracted using 2 mL of toluene and incubated at room temperature for 30 min. After separating the toluene phase, the absorbance was assessed at 520 nm using a UVmini-1240 spectrophotometer (Shimadzu, Kyoto, Japan).

The malondialdehyde (MDA) determination was calculated using an optimized method developed by Dhindsa and Matowe [61]. A leaf sample was homogenized in 5 mL of 0.1% trichloroacetic acid and centrifuged at 12,500× *g* for 20 min at 25 °C. Mixing 2 mL of supernatant with 2 mL of thiobarbituric acid-TCA. The reaction was stopped by immersing the tube in an ice bath for 10 min after 30 min of incubation at 90 °C. The chromogen was measured at 520 and 600 nm using a UVmini-1240 spectrophotometer (Shimadzu, Kyoto, Japan).

For enzyme extraction, 1 g of frozen leaf powder was homogenized in a cold mortar with 4 mL of 1 M phosphate buffer (pH 7.0) containing 0.1 mM Na-EDTA. The homogenate was centrifuged at 18,000× *g* for 15 min at 4 °C, and the supernatant was used for antioxidant enzyme activity assays [62].

Superoxide dismutase (SOD) activity was measured using the Cakmak and Marschner [63] technique. One unit of SOD activity was defined as the amount of enzyme required to inhibit nitro blue tetrazolium (NBT) by 50% at 25 °C. SOD activity was measured in min^−1^ mg of DM^−1^. The absorbance was measured at 650 nm using a UVmini-1240 spectrophotometer (Shimadzu, Kyoto, Japan).

The catalase (CAT) activity was determined by monitoring the decrease in absorbance at 240 nm over three minutes after adding H_2_O_2_. Aebi [64] reported that a 2 mL reaction mixture included 0.8 mL of 50 mM phosphate-buffered solution (pH 7.6), 0.1 mL of 100 mM H_2_O_2_, and 0.1 mL of enzyme extract.

To test glutathione reductase (GR), 0.1 mL of enzyme extract was homogenized in 0.7 mL of 50 mM phosphate-buffered solution (pH 7.6), 0.1 mM Na-EDTA, 0.5 mM oxidized glutathione (GSSG), and 0.12 mM NADPH [63,65]. The absorbance was measured using a Shimadzu UVmini-1240 spectrophotometer (Shimadzu, Kyoto, Japan) at a wavelength of 340 nm.

### 4.4. RNA Extraction and Quantitative Real-Time PCR (qRT-PCR)

The TRIzol method [66] was used to extract total RNA from shoot tissues of wheat seedlings, following the manufacturer’s instructions (Invitrogen, Waltham, MA, USA) and RNAase-free DNase I treatment (Thermo Fisher, Waltham, MA, USA). RNA concentration and quality were measured using a NanoDrop ND-1000 spectrophotometer (Thermo Fisher, Waltham, MA, USA). cDNA templates were created from total RNA samples via reverse transcription using the First Strand cDNA Synthesis Kit (Thermo Fisher, Waltham, MA, USA).

The transcript levels were measured using a CFX Connect™ 96 Real-Time PCR Detection System (Bio-Rad Laboratories GmbH, Hercules, CA, USA). qRT-PCR amplifications were conducted in a 15 µL reaction volume with 1.5 µL cDNA, 4 µL ddH2O, 1 µL forward (sense) and 1 µL reverse (antisense) primers, and 7.5 µL iTaq™ Universal SYBR^®^ Green Supermix (Bio-Rad Laboratories GmbH, Hercules, CA, USA). Each gene reaction was performed in triplicate using the following PCR protocol: 5 min at 94 °C, followed by 30 cycles of 30 s at 94 °C and 5 s at 65 °C. Melt curve analysis was subsequently performed from 75 °C to 95 °C to confirm amplification specificity. Quantitative real-time PCR (qRT-PCR) was performed to analyze the expression of salinity-responsive genes.

RNA samples were collected from shoot tissues of 7-day-old seedlings subjected to control (0 mM NaCl) and salinity treatment (150 mM NaCl). Gene-specific primers used for qRT-PCR are listed in Table S1. Primer efficiencies were evaluated prior to expression analysis and ranged between 90 and 110%. Primer specificity was verified by agarose gel electrophoresis (1.8%) and melt curve analysis. Each reaction was performed with three biological replicates and three technical replicates. *TaACTIN* (AB181991.1) was used as the internal reference gene, and relative gene expression levels were calculated using the 2^−^ΔΔCT method [67].

### 4.5. Statistical Analysis

ANOVA was performed on the obtained data using R software (V3.6.1, https://www.r-project.org/) to evaluate cultivar differences. Tukey’s HSD test at *p* < 0.05 was used to separate means using R software and the ‘glht’ function in the ‘multcomp’ package [68]. PCA was used to analyze the correlation matrix of 6 cultivars and their physiological and biochemical traits. Index values were calculated as the ratio between the salinity treatment and the control value for each trait (Index = Trait salt/Trait control). These index values were used for PCA and hierarchical clustering analyses to compare cultivar responses under salinity stress. The index values determined the correlation between response vectors and cultivars across the ordination space. A two-way heatmap clustering analysis (HCA) was conducted on the same dataset as the PCA. Pearson correlation was employed for distance, and the Euclidean algorithm was used to calculate the dissimilarity matrix. PCA and HCA were generated using the R software and the ‘prcomp’ function in the ‘factoextra’ package [69]. The heatmap function in the ‘pheatmap’ package, using R software, was used to cluster data hierarchically [70]. Random forest analysis was performed using the ‘randomForest’ package in R [71] to identify the most important physiological and biochemical traits associated with salinity tolerance. The model was constructed using 1000 trees, and trait importance was evaluated based on the mean decrease in accuracy and mean decrease in Gini index. Model performance was assessed using the out-of-bag (OOB) error estimation implemented in the randomForest algorithm.

## 5. Conclusions

This study demonstrates that early salinity responsiveness in wheat is associated with rapid biochemical and molecular alterations. Tolerant wheat cultivars exhibited stronger antioxidant responses, especially CAT, SOD, and GR, and osmoprotectant PRO pathways within early hours of salinity stress, which were associated with lower lipid peroxidation and improved early seedling establishment. However, salinity-sensitive cultivars are unable to activate the antioxidant defense system, resulting in higher lipid peroxidation from the early hours of seed germination to healthy seedlings. These early-hour defenses are associated with subsequent activation of *TaHKT1;4*, *TaP5CS*, *TaMYB*, and *TaDHN* expression during early seedling establishment under salinity. Moreover, the integration of multivariate and machine learning analysis highlights efficient indicators useful for broad screening. Our results improve the understanding of physiological and molecular responses associated with early salinity responses in wheat and provide efficient methods for the development of salinity-tolerant wheat cultivars for sustainable agriculture under changing climate conditions.

## Figures and Tables

**Table 1:** Analysis of variance (ANOVA) for biochemical, physiological, and gene expression traits in wheat genotypes under control and salt stress conditions. The significance levels of main effects [Cultivar (Cv), Environment (E)] and their interaction (C × E) are shown. Asterisks denote significance levels (* *p* < 0.05, ** *p* < 0.01, *** *p* < 0.001), and ns indicates non-significant differences.

Traits	Cv	E	Cv × E	Traits	Cv	E	Cv × E
MDA (1 h)	***	***	***	DM	***	***	***
MDA (3 h)	***	***	***	Chla	***	***	***
MDA (6 h)	***	***	***	Chlb	***	***	***
PRO (1 h)	***	ns	***	ChlT	***	***	***
PRO (3 h)	***	ns	***	MDA	***	***	***
PRO (6 h)	***	***	***	PRO	***	***	***
CAT (1 h)	***	***	***	CAT	***	ns	***
CAT (3 h)	***	***	***	SOD	***	***	***
CAT (6 h)	***	***	***	GR	***	***	***
SOD (1 h)	***	***	***	*TaPTF1*	***	***	***
SOD (3 h)	***	***	***	*TaDHN*	***	***	***
SOD (6 h)	***	***	***	*TaSRG*	***	***	***
GR (1 h)	***	***	***	*TaSC*	***	***	***
GR (3 h)	***	***	***	*TaPIMP*	***	***	***
GR (6 h)	***	***	***	*TaMIP*	***	**	***
GP	***	***	***	*TaHKT*	***	***	***
GSI	***	***	***	*TaGSK*	***	***	***
SH	***	***	***	*TaP5CS*	***	***	***
RL	***	***	***	*TaMYB*	***	***	***
FW	***	***	***				

Malondialdehyde (MDA), proline (PRO), catalase (CAT), superoxide dismutase (SOD), glutathione reductase (GR), chlorophyll a (Chla), chlorophyll b (Chlb), total chlorophyll (ChlT) pigments, shoot height (SH), root length (RL), fresh weight (FW), dry matter (DM), germination percentage (GP), germination speed index (GSI).

## Data Availability

Datasets are available on request.

## Supplementary Materials

The following supporting information can be downloaded at: https://www.mdpi.com/article/10.3390/plants15111593/s1, Figure S1: Early biochemical responses (1 h after salinity stress) in wheat cultivars; Figure S2: Early biochemical responses (3 h after salinity stress) in wheat cultivars; Figure S3: Early biochemical responses (6 h after salinity stress) in wheat cultivars; Figure S4: Growth and pigment responses of wheat cultivars under control and salt stress conditions; Figure S5: Late biochemical responses (7 d after salinity stress) in wheat cultivars; Figure S6: Expression of stress-related genes in wheat cultivars under control and salt stress conditions; Table S1: Primer sequence of genes used in the experiment; Table S2: Two-way analysis of variance (ANOVA) tables. Cultivar (Cv) and environment (E) interaction effects on measured traits; Table S3: Origin, development background, agronomic characteristics, and salinity tolerance classification of the wheat cultivars used in this study.

## Abbreviations

The following abbreviations are used in this manuscript:

MDA	Malondialdehyde
PRO	Proline
CAT	Catalase
SOD	Superoxide dismutase
GR	Glutathione reductase
Chla	Chlorophyll a
Chlb	Chlorophyll b
ChlT	Total chlorophyll
SH	Shoot height
RL	Root length
FW	Fresh weight
DM	Dry matter
GP	Germination percentage
GS	Germination speed
