# Supplementary material for: Initial Physiological and Molecular Adjustments Underpin Salinity Tolerance During Wheat Germination and Early Seedling Development
Source: Plants (Basel). 2026 May 22;15(11):1593. doi: 10.3390/plants15111593 (PMC13259275; doi:10.3390/plants15111593)
Supplement: Supplementary file 1 [file plants-15-01593-s001.zip › Supplementary files.pdf]

Supplementary Material

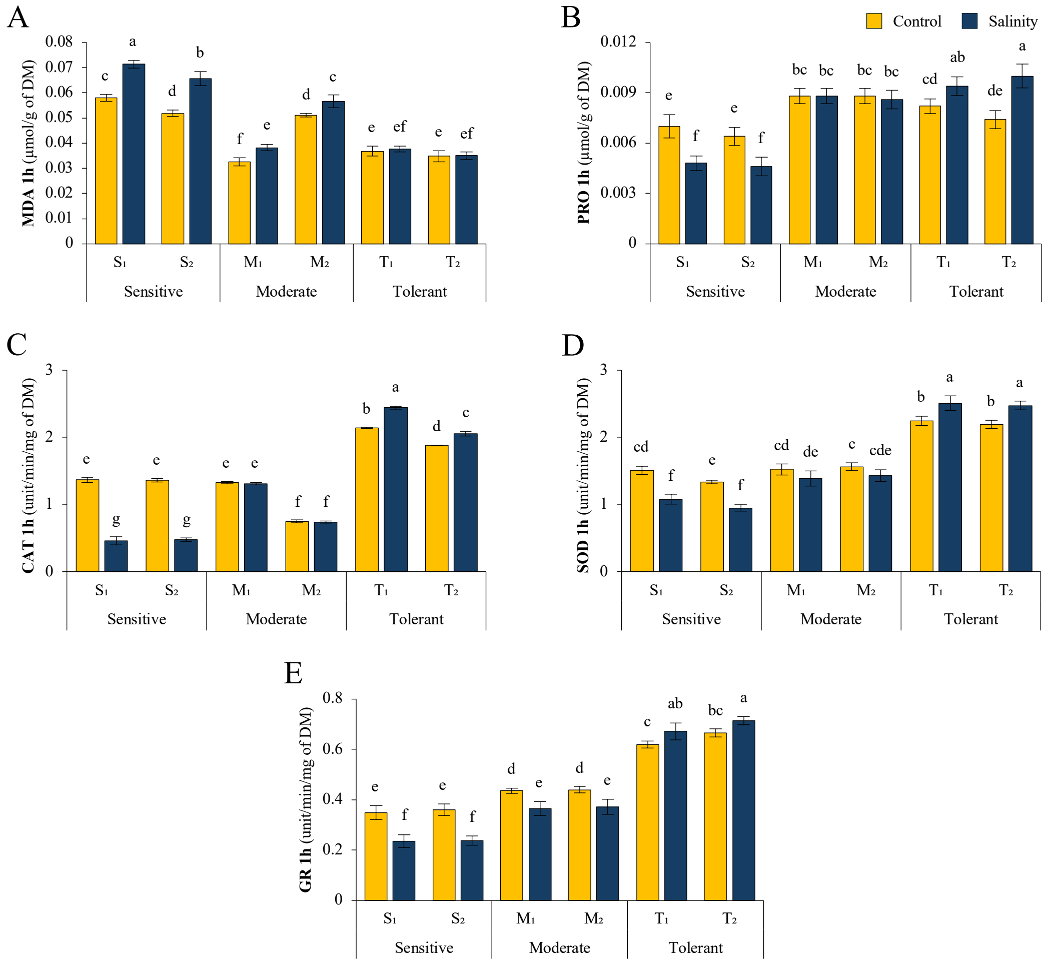

Figure S1. Early biochemical responses (1 h after salinity stress) in wheat cultivars. (A) Malondialdehyde (MDA), (B) proline (PRO), (C) catalase (CAT), (D) superoxide dismutase (SOD), and (E) glutathione reductase (GR) levels measured in sensitive (S1, S2), moderate (M1, M2), and tolerant (T1, T2) wheat cultivars under control (yellow, 0 mM NaCl) and salinity (blue, 150 mM NaCl) stress conditions for 1h. Bars represent mean ± SE (n = 5). Different letters above bars indicate significant differences among cultivar × treatment combinations (two-way ANOVA with Tukey's HSD, $P < 0.05$).

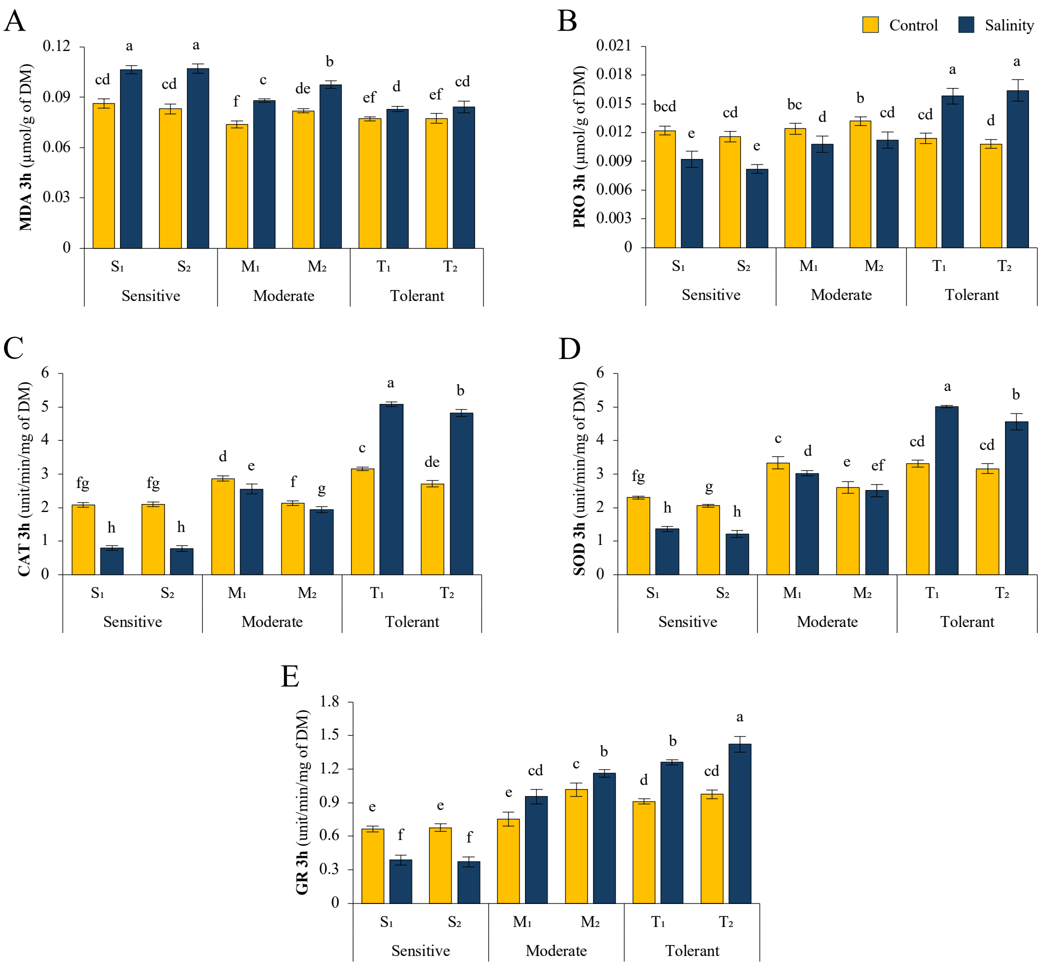

Figure S2. Early biochemical responses (3 h after salinity stress) in wheat cultivars. (A) Malondialdehyde (MDA), (B) proline (PRO), (C) catalase (CAT), (D) superoxide dismutase (SOD), and (E) glutathione reductase (GR) levels measured in sensitive (S1, S2), moderate (M1, M2), and tolerant (T1, T2) wheat cultivars under control (yellow, 0 mM NaCl) and salinity (blue, 150 mM NaCl) stress conditions for 1h. Bars represent mean ± SE (n = 5). Different letters above bars indicate significant differences among cultivar × treatment combinations (two-way ANOVA with Tukey's HSD, $P < 0.05$).

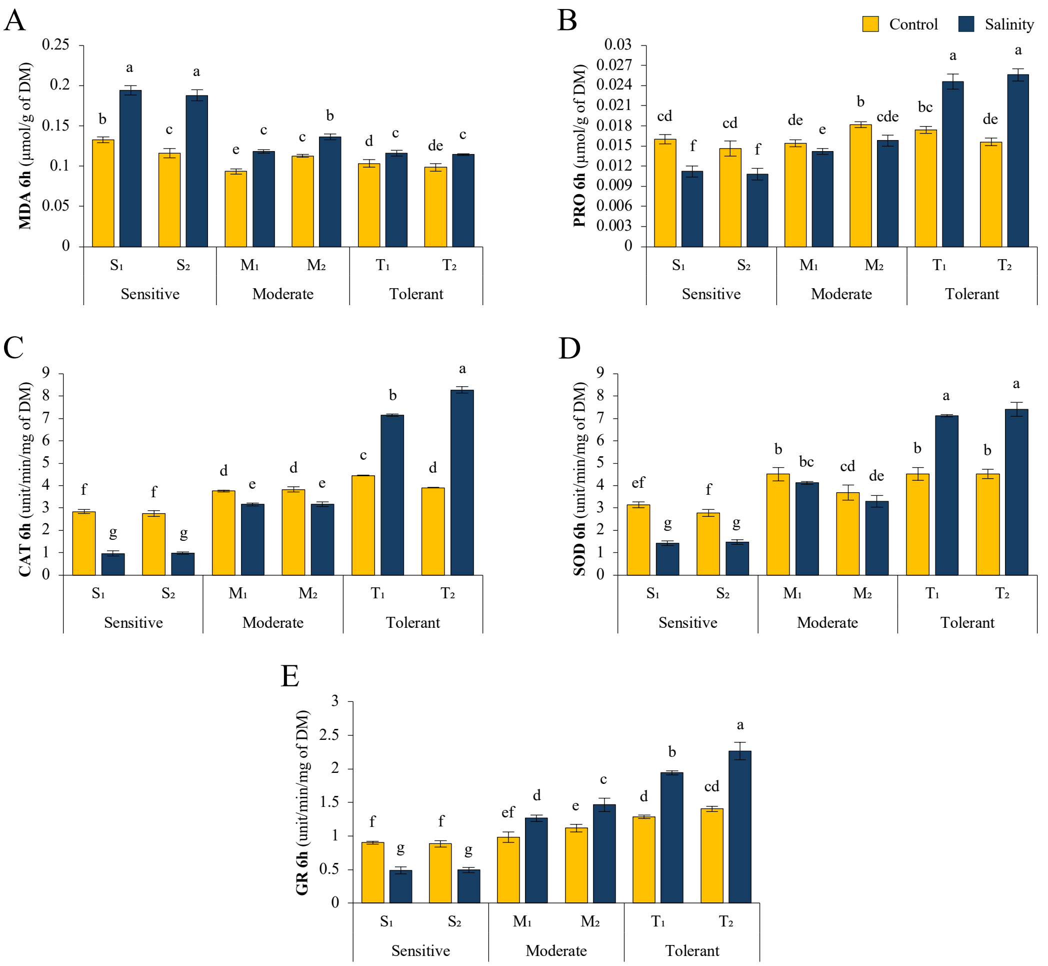

Figure S3. Early biochemical responses (6 h after salinity stress) in wheat cultivars. (A) Malondialdehyde (MDA), (B) proline (PRO), (C) catalase (CAT), (D) superoxide dismutase (SOD), and (E) glutathione reductase (GR) levels measured in sensitive (S1, S2), moderate (M1, M2), and tolerant (T1, T2) wheat cultivars under control (yellow, 0 mM NaCl) and salinity (blue, 150 mM NaCl) stress conditions for 1h. Bars represent mean ± SE (n = 5). Different letters above bars indicate significant differences among cultivar × treatment combinations (two-way ANOVA with Tukey's HSD, $P < 0.05$).

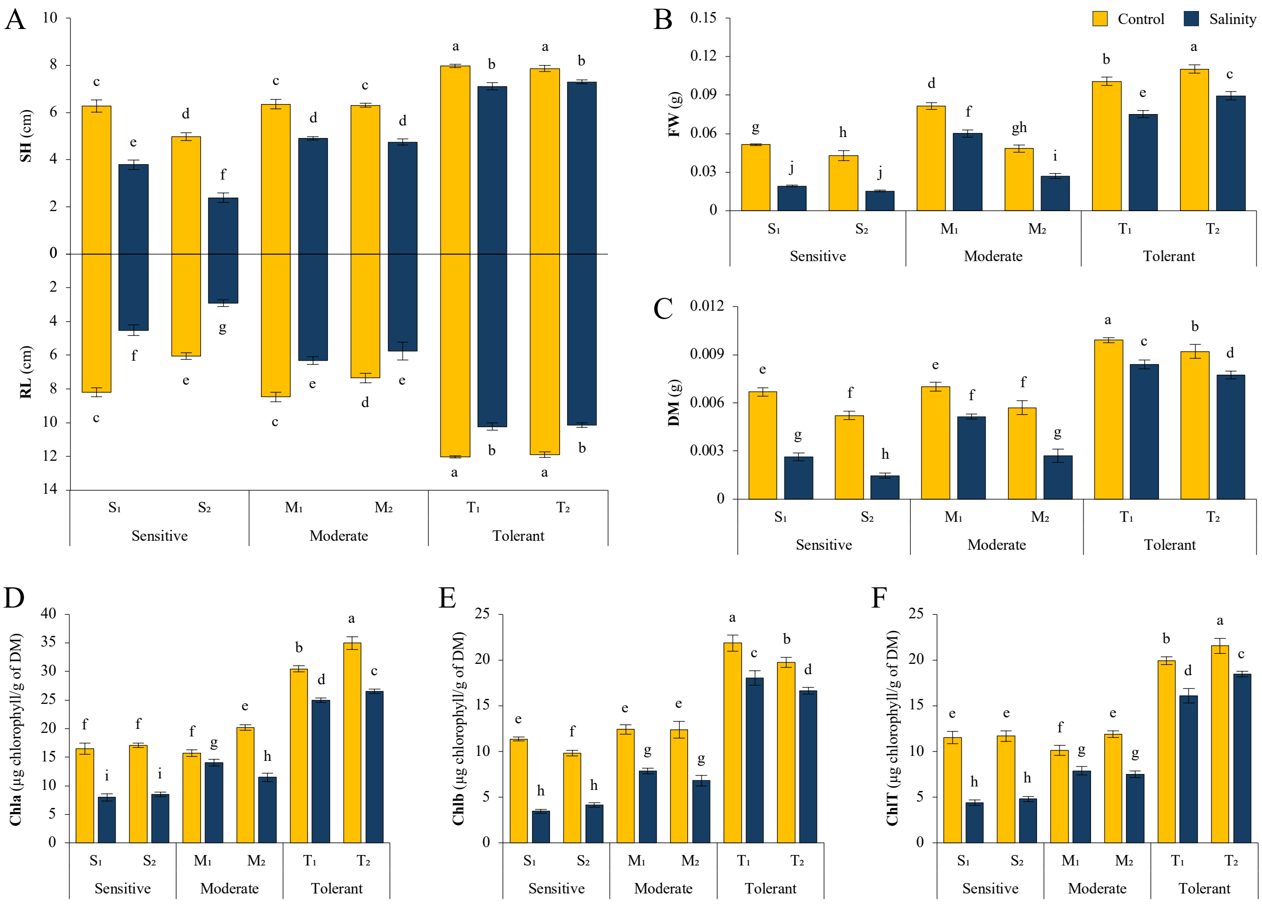

Figure S4. Growth and pigment responses of wheat cultivars under control and salt stress conditions. (A) Seedling heigh (SH) represent upper panel, root length (RL) represent lower panel, (B) fresh weight (FW), (C) dry matter (DM), (D) chlorophyll a (Chla), (E) chlorophyll b (Chlb), and (F) total chlorophyll (ChlT) contents in sensitive (S1, S2), moderate (M1, M2), and tolerant (T1, T2) wheat cultivars. Values are shown under control (yellow, 0 mM NaCl) and salt stress (blue, 150 mM NaCl) treatments. Bars represent mean ± SE (n = 5). Different letters above bars indicate significant differences among cultivar × treatment combinations (two-way ANOVA with Tukey's HSD, $P < 0.05$).

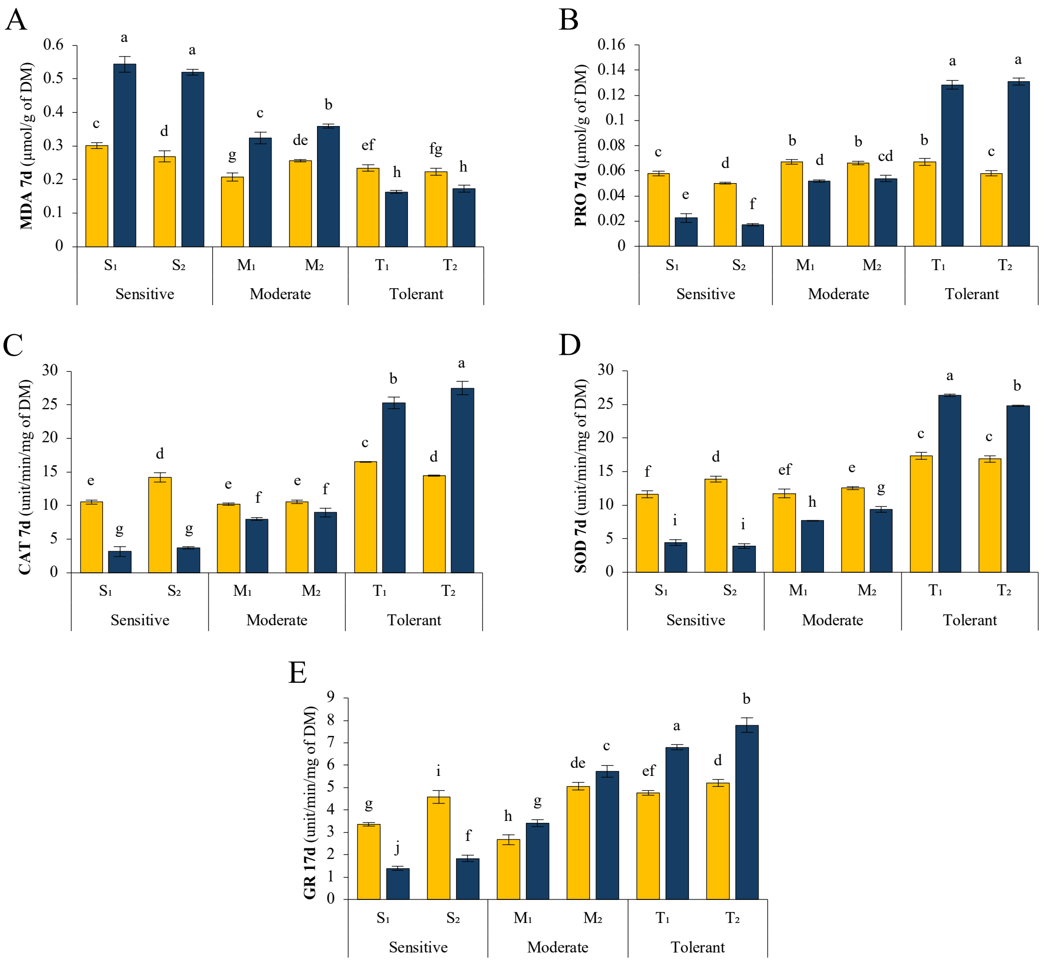

Figure S5. Late biochemical responses (7d after salinity stress) in wheat cultivars. (A) Malondialdehyde (MDA), (B) proline (PRO), (C) catalase (CAT), (D) superoxide dismutase (SOD), and (E) glutathione reductase (GR) levels measured in sensitive (S1, S2), moderate (M1, M2), and tolerant (T1, T2) wheat cultivars under control (yellow, 0 mM NaCl) and salinity (blue, 150 mM NaCl) stress conditions for 1h. Bars represent mean ± SE (n = 5). Different letters above bars indicate significant differences among cultivar × treatment combinations (two-way ANOVA with Tukey's HSD, $P < 0.05$).

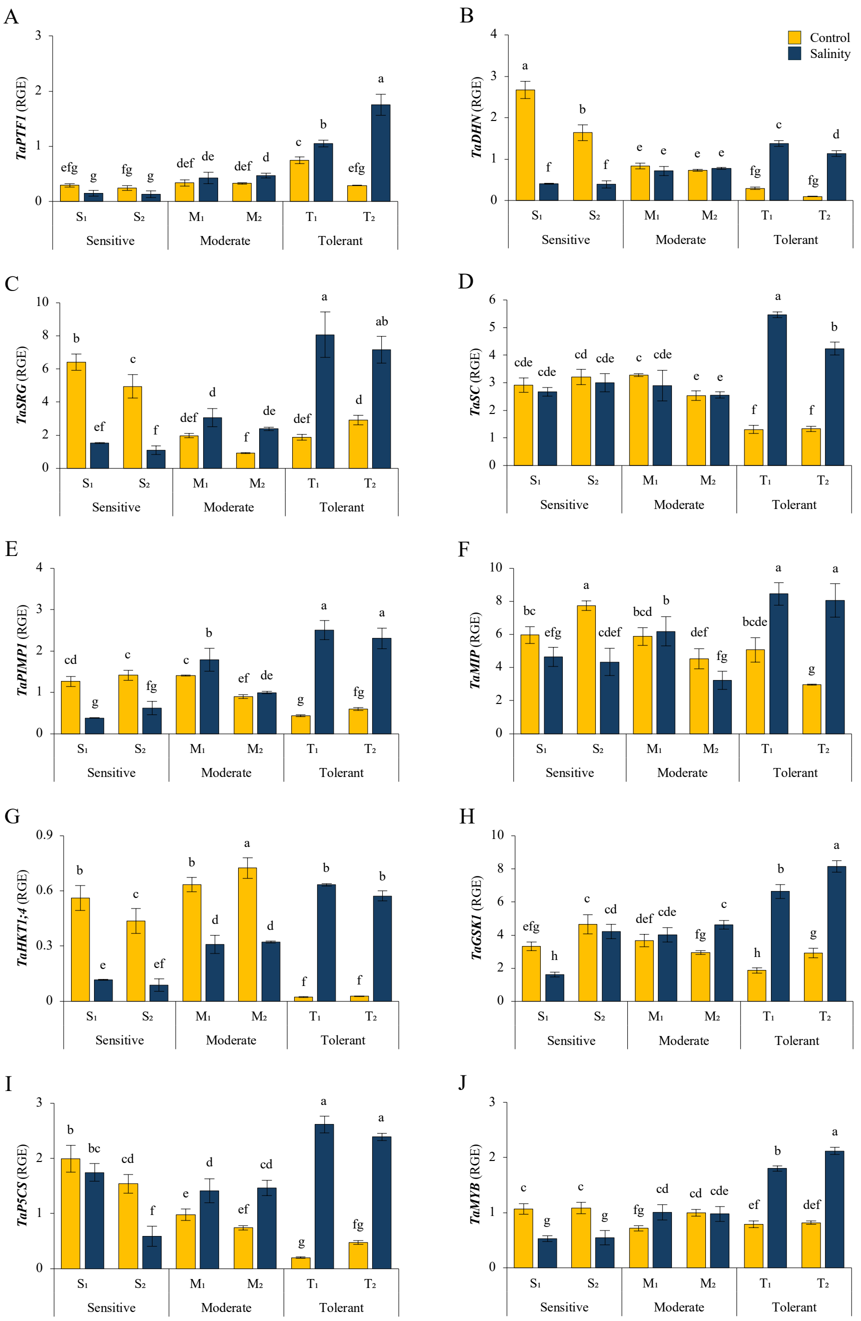

Figure S6. Expression of stress-related genes in wheat cultivars under control and salt stress conditions. Relative transcript levels of (A) *TaPTF1*, (B) *TaDHN*, (C) *TaSRG*, (D) *TaSC*, (E) *TaPIMP1*, (F) *TaMIP*, (G) *TaHKT1*;*4*, (H) *TaGSK1*, (I) *TaP5CS*, and (J) *TaMYB* in sensitive (S1, S2), moderate (M1, M2), and tolerant (T1, T2) wheat cultivars under control (yellow, 0 mM NaCl) and salt stress (blue, 150 mM NaCl) conditions. Gene expression was normalized against an internal reference (*TaACTIN*, AB181991.1) gene and expressed relative to the control condition. Bars represent mean ± SE (n = 5). Different letters above bars indicate significant differences among cultivar × treatment combinations (two-way ANOVA with Tukey's HSD, $P < 0.05$).

Table S1. Primer sequence of genes used in the experiment.

Gene name	Accession number	Forward Primer	Reverse Primer
TaPTF1	DQ979392.1	GAAGCGAAAGGGAGTGGAATATTG	CCAAAAGATGAGATGCTACCACTG
TaDHN	FN393741.1	GTCCCGACTTCCCGTAGTTG	CCTTGATGTTCTCGCCGGTA
TaSRG	DQ672342.1	CGGAGATTGCACAGCGAAATTAAG	AAGCTTCTTCATCCTCATCCTCTC
TaSC	AY956330.1	CACACACGGACACCAAGTAATC	CCAGTATGTCAACCCGCTTATCAA
TaPIMP1	EU004200.1	TTCAGTCTCCTTATCTGGCATCTG	GCGACCAGAATGCCTAATATGTTC
TaHKT1;4	HG934161.1	AGCAAGCTGAAGTTGAGGGG	AGAGTTGTGACAGAGCCGTG
TaGSK1	AF525086.1	CATGGGTGGTTTGTTACATCGG	GACAATCTCAAACTCCTGGGGT
TaP5CS	KM523670.1	GAAGGCTCTTATGGGTGTACTCAA	TAAAAGACCTTCAACACCCACAGG
TaACTIN	AB181991.1	CAAAGAGATCACGGCCCTTG	CGGCATTGTCCACATGAAGT

Table S2. Two-way analysis of variance (ANOVA) tables. Cultivar (Cv) and environment (E) interaction effects on measured traits. Asterisks indicate significant differences (*** $P < 0.001$, ** $P < 0.01$, * $P < 0.05$, ns = non-significant).

MDA 1h	Df	Sum Sq	Mean Sq	F value	Pr(>F)	
Cv	5	0.008722	0.0017444	576.67	< 2e-16	***
E	1	0.000647	0.0006468	213.82	< 2e-16	***
Cv x E	5	0.000437	0.0000873	28.87	2.15E-13	***
Residuals	48	0.000145	0.000003			

MDA 3h	Df	Sum Sq	Mean Sq	F value	Pr(>F)	
Cv	5	0.0028705	0.0005741	102.22	<2e-16	***
E	1	0.0031393	0.0031393	558.92	<2e-16	***
Cv x E	5	0.0006473	0.0001295	23.05	1.01E-11	***
Residuals	48	0.0002696	0.0000056			

MDA 6h	Df	Sum Sq	Mean Sq	F value	Pr(>F)	
Cv	5	0.031255	0.006251	356.18	<2e-16	***
E	1	0.018445	0.018445	1051	<2e-16	***
Cv x E	5	0.007876	0.001575	89.75	<2e-16	***
Residuals	48	0.000842	0.000018			

PRO 1h	Df	Sum Sq	Mean Sq	F value	Pr(>F)	
Cv	5	1.25E-04	2.50E-05	85.669	< 2e-16	***
E	1	7.00E-08	6.70E-08	0.229	6.35E-01	ns
Cv x E	5	4.07E-05	8.15E-06	27.931	3.85E-13	***
Residuals	48	1.40E-05	2.92E-07			

PRO 3h	Df	Sum Sq	Mean Sq	F value	Pr(>F)	
Cv	5	0.0001139	2.28E-05	47.15	<2e-16	***
E	1	0	0.00E+00	0	1.00E+00	ns
Cv x E	5	0.0001946	3.89E-05	80.52	<2e-16	***
Residuals	48	0.0000232	4.80E-07			

PRO 6h	Df	Sum Sq	Mean Sq	F value	Pr(>F)	
Cv	5	0.0006297	1.26E-04	207.02	<2e-16	***
E	1	0.0000104	1.04E-05	17.12	1.40E-04	***
Cv x E	5	0.0004809	9.62E-05	158.1	<2e-16	***
Residuals	48	0.0000292	6.10E-07			

CAT 1h	Df	Sum Sq	Mean Sq	F value	Pr(>F)	
Cv	5	20.186	4.037	4868.9	<2e-16	***
E	1	0.745	0.745	898.8	<2e-16	***
Cv x E	5	3.561	0.712	858.9	<2e-16	***
Residuals	48	0.04	0.001			

CAT 3h	Df	Sum Sq	Mean Sq	F value	Pr(>F)	
Cv	5	66.92	13.384	1808	< 2e-16	***
E	1	0.36	0.361	48.81	7.71E-09	***
Cv x E	5	28.81	5.761	778.27	< 2e-16	***
Residuals	48	0.36	0.007			

CAT 6h	Df	Sum Sq	Mean Sq	F value	Pr(>F)	
Cv	5	168.02	33.6	4412.3	<2e-16	***
E	1	1.99	1.99	260.8	<2e-16	***
Cv x E	5	82.58	16.52	2168.4	<2e-16	***
Residuals	48	0.37	0.01			

SOD 1h	Df	Sum Sq	Mean Sq	F value	Pr(>F)	
Cv	5	14.383	2.8765	511.36	< 2e-16	***
E	1	0.126	0.1258	22.36	2.02E-05	***
Cv x E	5	1.176	0.2352	41.8	2.30E-16	***
Residuals	48	0.27	0.0056			

SOD 3h	Df	Sum Sq	Mean Sq	F value	Pr(>F)	
Cv	5	54.52	10.905	597.35	< 2e-16	***
E	1	0.34	0.342	18.71	7.66E-05	***
Cv x E	5	15.99	3.198	175.17	< 2e-16	***
Residuals	48	0.88	0.018			

SOD 6h	Df	Sum Sq	Mean Sq	F value	Pr(>F)	
Cv	5	139.62	27.924	592.2	< 2e-16	***
E	1	1.19	1.186	25.15	7.65E-06	***
Cv x E	5	48.97	9.795	207.72	< 2e-16	***
Residuals	48	2.48	0.047			

GR 1h	Df	Sum Sq	Mean Sq	F value	Pr(>F)	
Cv	5	1.4691	0.29381	577.14	< 2e-16	***
E	1	0.031	0.03101	60.91	4.35E-10	***
Cv x E	5	0.0756	0.01512	29.71	1.29E-13	***
Residuals	48	0.0244	0.00051			

GR 3h	Df	Sum Sq	Mean Sq	F value	Pr(>F)	
Cv	5	4.413	0.8827	399.36	<2e-16	****
E	1	0.131	0.1311	59.33	6.22E-10	****
Cv x E	5	1.254	0.2509	113.5	<2e-16	****
Residuals	48	0.106	0.0022			

GR 6h	Df	Sum Sq	Mean Sq	F value	Pr(>F)	
Cv	5	11.019	2.2038	549	<2e-16	***
E	1	0.751	0.7508	187	<2e-16	***
Cv x E	5	3.51	0.7019	174.9	<2e-16	***
Residuals	48	0.193	0.004			

GP	Df	Sum Sq	Mean Sq	F value	Pr(>F)	
Cv	5	11658	2332	594	<2e-16	***
E	1	25297	25297	6445.1	<2e-16	***
Cv x E	5	11655	2331	593.9	<2e-16	***
Residuals	48	188	4			

GS	Df	Sum Sq	Mean Sq	F value	Pr(>F)	
Cv	5	1.666	0.333	71.38	<2e-16	***
E	1	6.733	6.733	1442.89	<2e-16	***
Cv x E	5	1.665	0.333	71.38	<2e-16	***
Residuals	48	0.224	0.005			

MDA 7d	Df	Sum Sq	Mean Sq	F value	Pr(>F)	
Cv	5	0.4568	0.09136	616.8	<2e-16	***
E	1	0.1449	0.14494	978.5	<2e-16	***
Cv x E	5	0.2384	0.04767	321.9	<2e-16	***
Residuals	48	0.0071	0.00015			

Table S2. Continue

PRO 7d	Df	Sum Sq	Mean Sq	F value	Pr(>F)	
Cv	5	0.03585	0.007171	1328	<2e-16	***
E	1	0.00062	0.000621	115	2.46E-14	***
Cv x E	5	0.02891	0.005782	1071	<2e-16	***
Residuals	48	0.00026	0.000005			

CAT 7d	Df	Sum Sq	Mean Sq	F value	Pr(>F)	
Cv	5	2059.9	412	1417.896	<2e-16	***
E	1	0	0	0.061	8.06E-01	ns
Cv x E	5	1047.1	209.4	0.3	<2e-16	***
Residuals	48	13.9				

SOD 7d	Df	Sum Sq	Mean Sq	F value	Pr(>F)	
Cv	5	1949.5	389.9	2417.1	<2e-16	***
E	1	22.9	22.9	142	6.00E-16	***
Cv x E	5	781.4	156.3	968.8	<2e-16	***
Residuals	48	7.7	0.2			

GR 7d	Df	Sum Sq	Mean Sq	F value	Pr(>F)	
Cv	5	146.44	29.289	788.66	< 2e-16	***
E	1	0.74	0.737	19.83	5.04E-05	***
Cv x E	5	57.32	11.463	308.68	< 2e-16	***
Residuals	48	1.78	0.037			

SH	Df	Sum Sq	Mean Sq	F value	Pr(>F)	
Cv	5	113.78	22.76	931.09	<2e-16	***
E	1	37.67	37.67	1541.22	<2e-16	***
Cv x E	5	8.45	1.69	69.15	<2e-16	***
Residuals	48	1.17	0.02			

RL	Df	Sum Sq	Mean Sq	F value	Pr(>F)	
Cv	5	363.4	72.68	1049.14	< 2e-16	***
E	1	82.9	82.88	1196.41	< 2e-16	***
Cv x E	5	9.1	1.82	26.34	1.07E-12	***
Residuals	48	3.3	0.07			

FW	Df	Sum Sq	Mean Sq	F value	Pr(>F)	
Cv	5	0.04538	0.009076	1308.735	<2e-16	***
E	1	0.00928	0.009275	1337.473	<2e-16	***
Cv x E	5	0.00025	0.00005	7.279	3.84E-05	***
Residuals	48	0.00033	0.000007			

DM	Df	Sum Sq	Mean Sq	F value	Pr(>F)	
Cv	5	2.81E-04	5.62E-05	643.2	<2e-16	***
E	1	1.01E-04	1.01E-04	1158.1	<2e-16	***
Cv x E	5	1.66E-05	3.31E-06	37.9	1.49E-15	***
Residuals	48	0.0000042	9.00E-08			

Chla	Df	Sum Sq	Mean Sq	F value	Pr(>F)	
Cv	5	3249	649.8	1587.56	<2e-16	***
E	1	709	708.7	1731.31	<2e-16	***
Cv x E	5	101	20.2	49.27	<2e-16	***
Residuals	48	20	0.4			

Chlb	Df	Sum Sq	Mean Sq	F value	Pr(>F)	
Cv	5	1564.8	313	1044.21	<2e-16	***
E	1	389.7	389.7	1300.16	<2e-16	***
Cv x E	5	35.6	7.1	23.79	6.00E-12	***
Residuals	48	14.4	0.3			

ChlT	Df	Sum Sq	Mean Sq	F value	Pr(>F)	
Cv	5	1443.3	288.65	1063.05	< 2e-16	***
E	1	316	315.97	1163.66	< 2e-16	***
Cv x E	5	50.1	10.03	36.93	2.42E-15	***
Residuals	48	13	0.27			

TaPTF1	Df	Sum Sq	Mean Sq	F value	Pr(>F)	
Cv	5	6.308	1.2615	213.6	<2e-16	***
E	1	1.264	1.2636	214	<2e-16	***
Cv x E	5	4.463	0.8926	151.2	<2e-16	***
Residuals	48	0.283	0.0059			

TaDHN	Df	Sum Sq	Mean Sq	F value	Pr(>F)	
Cv	5	5.423	1.085	111.77	<2e-16	***
E	1	0.882	0.882	90.91	1.18E-12	***
Cv x E	5	21.51	4.302	443.33	<2e-16	***
Residuals	48	0.466	0.01			

TaSRG	Df	Sum Sq	Mean Sq	F value	Pr(>F)	
Cv	5	93.61	18.72	59.32	<2e-16	***
E	1	7.57	7.57	24	1.14E-05	***
Cv x E	5	238.83	47.77	151.34	<2e-16	***
Residuals	48	15.15	0.32			

TaSC	Df	Sum Sq	Mean Sq	F value	Pr(>F)	
Cv	5	4.54	0.907	14.85	8.43E-09	***
E	1	16.39	16.385	268.28	<2e-16	***
Cv x E	5	48.67	9.733	159.36	<2e-16	***
Residuals	48	2.93	0.061			

TaPIMP	Df	Sum Sq	Mean Sq	F value	Pr(>F)	
Cv	5	5.305	1.061	50.16	<2e-16	***
E	1	2.75	2.75	130.04	2.89E-15	***
Cv x E	5	19.132	3.826	180.92	<2e-16	***
Residuals	48	1.015	0.021			

TaMIP	Df	Sum Sq	Mean Sq	F value	Pr(>F)	
Cv	5	47.63	9.525	22.367	1.66E-11	***
E	1	3.15	3.148	7.392	0.00909	**
Cv x E	5	127.91	25.583	60.075	<2e-16	***
Residuals	48	20.44	0.426			

TaHKT	Df	Sum Sq	Mean Sq	F value	Pr(>F)	
Cv	5	0.5325	0.1065	68.94	<2e-16	***
E	1	0.0556	0.0556	36.02	2.50E-07	***
Cv x E	5	3.0865	0.6173	399.62	<2e-16	***
Residuals	48	0.0741	0.0015			

TaGSK	Df	Sum Sq	Mean Sq	F value	Pr(>F)	
Cv	5	50.13	10.03	83.83	<2e-16	***
E	1	40.83	40.83	341.44	<2e-16	***
Cv x E	5	99.83	19.97	166.95	<2e-16	***
Residuals	48	5.74	0.12			

TaP5CS	Df	Sum Sq	Mean Sq	F value	Pr(>F)	
Cv	5	4.483	0.897	42.42	<2e-16	***
E	1	7.637	7.637	361.32	<2e-16	***
Cv x E	5	20.265	4.053	191.77	<2e-16	***
Residuals	48	1.014	0.021			

TaMYB	Df	Sum Sq	Mean Sq	F value	Pr(>F)	
Cv	5	3.942	0.7884	100.2	<2e-16	***
E	1	0.945	0.9447	120	1.17E-14	***
Cv x E	5	7.497	1.4993	190.5	<2e-16	***
Residuals	48	0.378	0.0079			

Table S3. Origin, development background, agronomic characteristics, and salinity tolerance classification of the wheat cultivars used in this study.

Cultivar	Salinity group	Origin country	Development method	Reported agronomic background
Sonmez-01 (S_1)	Sensitive	Türkiye	Hybridization	Drought tolerant; yield: 6000–6500 kg/ha; resistant to yellow rust disease
Esperia (S_2)	Sensitive	Italy	Introduction	Cold tolerant; yield: 5000–7500 kg/ha; resistant to brown rust disease
Bezostoja (M_1)	Moderate	Russia	Introduction	Yield: 3500–4000 kg/ha; resistant to yellow rust disease
Gerek-79 (M_2)	Moderate	Türkiye	Hybridization	Drought tolerant; yield: 4500–5000 kg/ha; resistant to yellow rust disease
Ikizce-96 (T_1)	Tolerant	Türkiye	Hybridization	Drought tolerant; yield: 3000–3500 kg/ha; sensitive to yellow rust disease
Demir-2000 (T_2)	Tolerant	Türkiye	Hybridization	Cold and drought tolerant; yield: 5000–6000 kg/ha; resistant to yellow rust disease
